# 3-Hydroxy-3-methylglutaryl coenzyme A reductase genes from *Glycine max* regulate plant growth and isoprenoid biosynthesis

**DOI:** 10.1038/s41598-023-30797-4

**Published:** 2023-03-08

**Authors:** Shuai Wang, Yumei Feng, Yin Lou, Jingping Niu, Congcong Yin, Jinzhong Zhao, Weijun Du, Aiqin Yue

**Affiliations:** 1 College of Agronomy, Shanxi Agricultural University, Taigu, 030801 Shanxi China; 2 College of Forestry, Shanxi Agricultural University, Taigu, 030801 Shanxi China; 3 College of Life Sciences, Shanxi Agricultural University, Taigu, 030801 Shanxi China; 4 Department of Basic Sciences, Shanxi Agricultural University, Taigu, 030801 Shanxi China

**Keywords:** Secondary metabolism, Gene regulation, Gene expression, Transgenic plants, Bioinformatics

## Abstract

Isoprenoids, a large kind of plant natural products, are synthesized by the mevalonate (MVA) pathway in the cytoplasm and the 2-C-methyl-d-erythritol 4-phosphate (MEP) pathway in plastids. As one of the rate-limiting enzymes in the MVA pathway of soybean (*Glycine max*), 3-hydroxy-3-methylglutaryl coenzyme A reductase (HMGR) is encoded by eight isogenes (*GmHMGR1–GmHMGR8*). To begin, we used lovastatin (LOV), a specific inhibitor of GmHMGR, to investigate their role in soybean development. To further investigate, we overexpressed the *GmHMGR4* and *GmHMGR6* genes in *Arabidopsis thaliana*. The growth of soybean seedlings, especially the development of lateral roots, was inhibited after LOV treatment, accompanied by a decrease in sterols content and *GmHMGR* gene expression. After the overexpression of *GmHMGR4* and *GmHMGR6* in *A. thaliana*, the primary root length was higher than the wild type, and total sterol and squalene contents were significantly increased. In addition, we detected a significant increase in the product tocopherol from the MEP pathway. These results further support the fact that *GmHMGR1–GmHMGR8* play a key role in soybean development and isoprenoid biosynthesis.

## Introduction

Isoprenoids or isoprenoid derivatives, including chlorophyll, carotenoids, sterols, tocopherols, terpenoids and so on, are a kind of important natural products in plants. They are crucial for plant growth, development, disease-resistant biotic, abiotic stress and so on^1–5^. The common biosynthetic intermediates for the production of isoprenoids, isopentenyl pyrophosphate (IPP) and its isomer dimethylallyl pyrophosphate (DMAPP), are produced via the mevalonate (MVA) and 2-C-methyl-d-erythritol 4-phosphate (MEP) pathways^6,7^. Sterols, saponins and triterpenes are produced by the mevalonate (MVA) pathways, while chlorophyll, carotenoids, tocopherols, and diterpenoids are synthesized by the MEP pathway^8–10^. Many studies have reported on a connection between the MVA and MEP pathways^11–13^. Yet, the crosstalk between the two pathways in soybean (*Glycine max*) is still unclear.

3-Hydroxy-3-methylglutaryl coenzyme A reductase (HMGR) is responsible for catalyzing 3-hydroxy-3-methylglutaryl-CoA (HMG-CoA) convert to MVA via two successive hydride transfers, using two molecules of NADPH. Then the obtained MVA further produced IPP by several enzymatic reaction^6,14,15^. Numerous studies have indicated that HMGR is the major rate-limiting enzyme regulating carbon flux through the MVA pathway^7,16,17^. At present, *HMGR* genes have been cloned and characterized from a number of plants, such as *Arabidopsis thaliana*^4,18,19^, *Salvia miltiorrhiza*^20^, *Gossypium*^21^, *Ginkgo biloba*^22^, *Medicago truncatula*^3^, *Populus trichocarpa*^23^, *Cyanotis arachnoidea*^24^, and *Hevea brasiliensis*^25^. Many studies have indicated that *HMGR* gene(s) overexpression or silence significantly affected isoprenoids content and plant growth. According to Kim et al.^26^, the expression of *PgHMGR* had a significant correlation with ginsenoside content in *Panax ginseng.* In addition, the overexpression of *PgHMGR1* in *A. thaliana* and *P. ginseng* significantly enhanced triterpenoid production in Kim’s paper^26^. The loss of the function mutation of *AtHMGR1* gene in *A. thaliana* led to dwarfing, early senescence and male sterility^4^. Overexpression of the *SmHMGR2* gene in *S. miltiorrhiza* upregulated the formation of tanshinone and squalene in hairy roots^20^. The content of total sterol in tobacco seeds was increased by the overexpression of the *HbHMGR* gene^27^.

HMGR in plants is generally encoded by a multigenic family^4,16,21,26^, such as there are three *HMGR* genes in *Solanum tuberosum*^28^ and two *HMGR* genes in *A. thaliana*^4^. Therefore, it is difficult to knock out *HMGR* genes to investigate their function. Lovastatin (LOV), a specific inhibitor of plant HMGR, has been used to verify the function of HMGR in *A. thaliana*^29,30^, *Trigonella foenum-graecuml*^31^, *Ocimum kilimandscharicum*^32^, *Spike Lavender*^13^ and so on. In our previou study, we had cloned eight *GmHMGR* genes (named *GmHMGR1*, *GmHMGR2*, *GmHMGR3*, *GmHMGR4*, *GmHMGR5*, *GmHMGR6*, *GmHMGR7*, and *GmHMGR8*) from soybean, which displayed different spatial and temporal gene expression patterns^33^. A recent study showed that the *GmHMGR1* gene plays an important role in triggering nodule formation^34^. However, the function of *GmHMGR* genes during soybean growth and isoprenoid biosynthesis has not been studied. For the reason that the L-domain (the HMG-CoA binding site) in C-terminus is conservative among HMGR, LOV is expected to exert a similar effect in soybean^14,15^. So, to investigate the function of *GmHMGR* genes in soybean development and isoprenoid metabolism, we first observed that soybean seedlings after LOV treatment presented a retarded primary root growth tendency. Subsequently, we conducted additional researches to find out the reason for these phenomena by measuring the content of isoprenoids and the expression of related genes. In addition, overexpression of *GmHMGR4* and *GmHMGR6* in *A. thaliana* was to explore the mechanism of *GmHMGR* in facilitating *A. thaliana* growth and to test whether the metabolic pathways of isoprenoids were affected.

## Results

### Multiple sequence alignments and molecular docking of GmHMGR proteins

**Figure 1:**
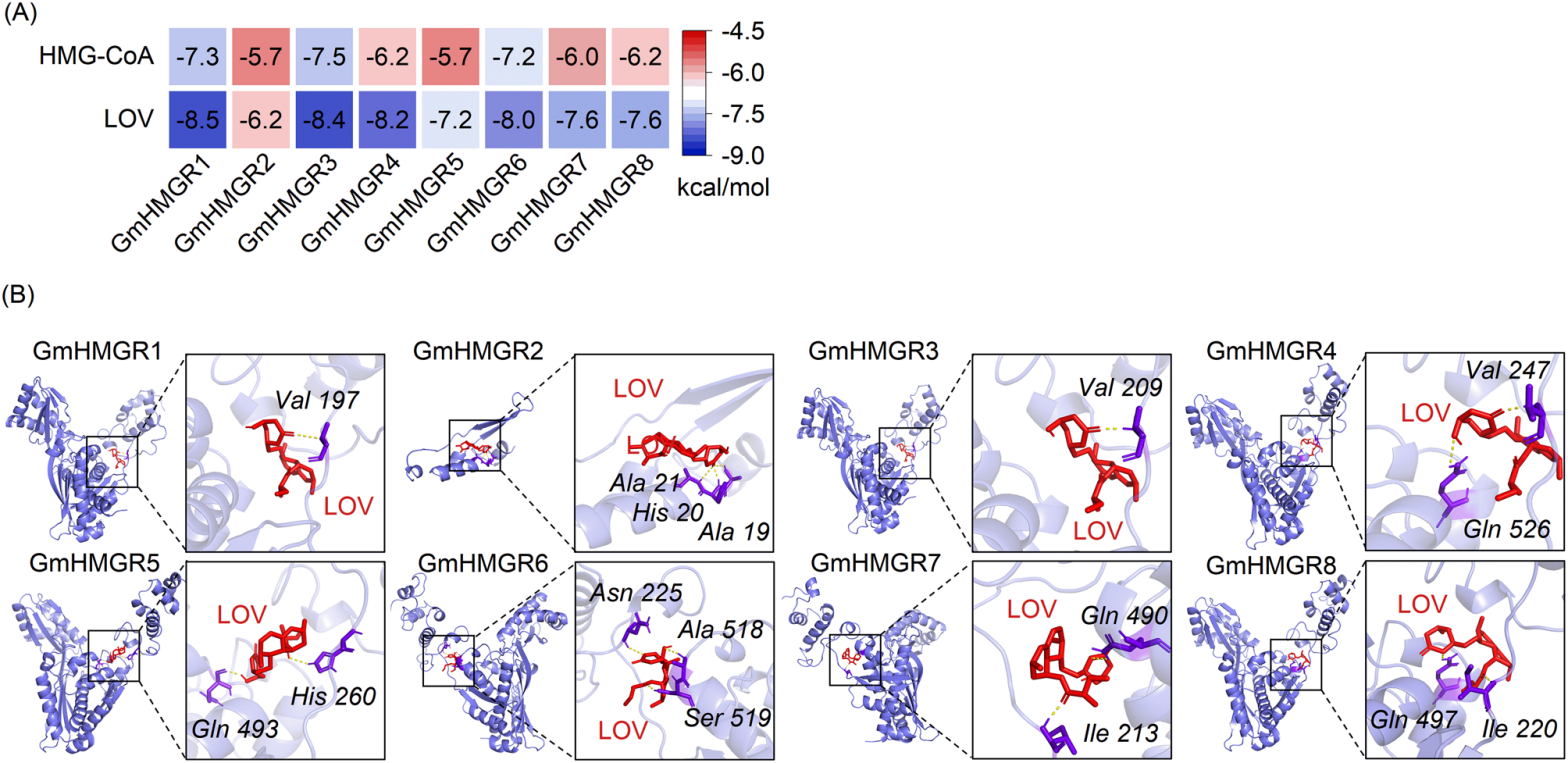
Docking analysis of GmHMGR proteins binding with LOV. (**A**) The docking binding energies of GmHMGR proteins with HMG-CoA and LOV. (**B**) Docking analysis visualization of GmHMGR binding with LOV. Amino acids that form hydrogen bonds are identified in purple while hydrogen bonds are shown in yellow.

Targeting eight *HMGR* genes in soybean, we aligned the eight GmHMGR proteins’ amino acid sequences and analysed their conserved elements (Supplementary Fig. S1). GmHMGR proteins, except for GmHMGR2, contained HMG-CoA and NADP(H) binding motifs. However, GmHMGR2 only had the NADP(H) binding motif II. To investigate whether LOV interacts with GmHMGR proteins, we examined the binding energies from the interaction of GmHMGR proteins’ docking sites with LOV and HMG-CoA by using AutoDockVina. The conformers with the lowest binding energy were chosen for molecular docking analysis. As shown in Fig. 1A, the binding energy of GmHMGR1-GmHMGR8 to LOV was − 8.5, − 6.2, − 8.4, − 8.2, − 7.2, − 8.0, − 7.6 and − 7.6 kcal∙mol^−1^, respectively, and all less than that of GmHMGR proteins to HMG-CoA. The above results suggested that LOV might suppress GmHMGR1-GmHMGR8 by competing with HMG-CoA. As is indicated in Fig. 1B, LOV could bind to the catalytic core of eight GmHMGR proteins respectively and form hydrogen bonds. The docking results showed that the structural features of soybean HMGR isoforms were consistent with LOV binding.

### Inhibitive effects of LOV on soybean seedling growth

**Figure 2:**
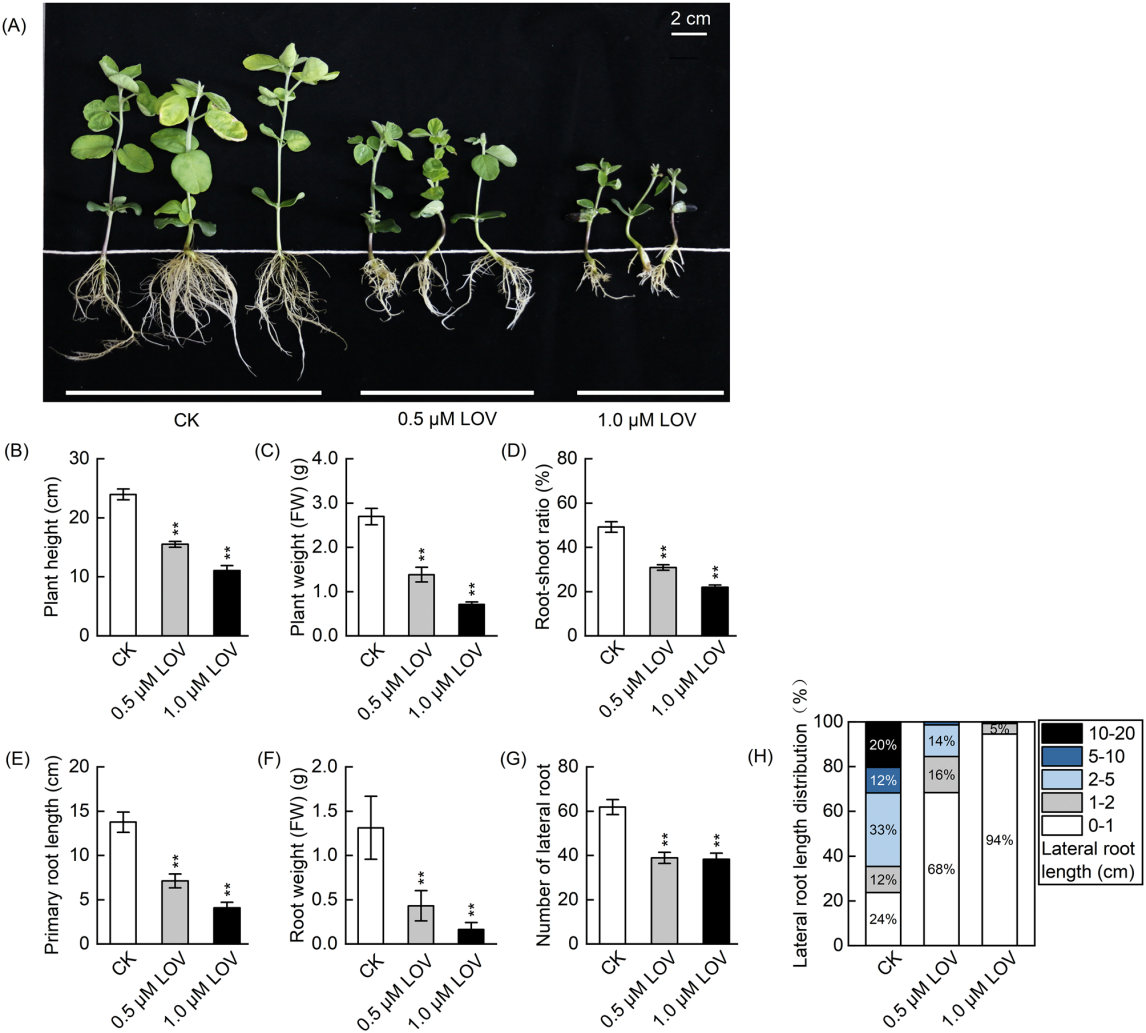
Effect of LOV on the growth of soybean seedlings. (**A**) Images of soybean seedlings after LOV treatment. (**B**) Plant height, (**C**) plant weight (FW), (**D**) root-shoot ratio, (**E**) primary root length, (**F**) root weight (FW), and (**G**) the number of lateral roots of soybean seedlings after LOV treatment and corresponding blank control (CK). Values are means ± standard error (n = 10); the symbol * denotes significant difference from CK at *p* < 0.05; ** at *p* < 0.01 by Student’s t-tests. (**H**) Differences in lateral root length distribution of soybean seedlings after LOV treatment and corresponding CK. Counting lateral roots in soybean seedlings (n > 15) per LOV treatment.

Based on preliminary experiments, we used 0.5 and 1.0 µM LOV to process the soybean seedlings to further investigate the mechanism of *GmHMGR* in facilitating soybean growth and isoprenoid biosynthesis. As shown in Fig. 2A, the use of LOV distinctly inhibited the growth of the soybean seedlings, and this effect was dose-dependent. In the meantime, soybean seedling roots exhibited a significant inhibition to HMGR activity (Supplementary Fig. S2). 0.5 and 1.0 µM LOV significantly decreased plant height (Fig. 2B), plant weight (Fig. 2C) and root-shoot ratio (Fig. 2D). For example, the plant height was reduced by 35.25% and 53.89% after 0.5 and 1.0 µM LOV treatments, respectively. In addition, the results showed that LOV effectively retarded primary root and lateral root growth (Fig. 2E–G). The primary root length decreased dramatically by 70.31% after 1.0 µM LOV treatment (Fig. 2E), and it was 48.23% shorter in 0.5 µM LOV-treated soybean seedlings. After LOV treatment, the number of lateral roots of soybean seedlings decreased by over 37.10% (Fig. 2G). LOV also strongly changed the distribution of different lateral root lengths. After 1.0 µM LOV treatment, 94.50% of the lateral roots did not exceed 1 cm in length, which showed obvious lateral root morphological deficits (Fig. 2A,H). These results demonstrated that the use of the HMGR-specific inhibitor (LOV) in soybeans was an effective approach to studying the MVA pathway.

### Effects of LOV on lateral root structure of soybean seedlings

**Figure 3:**
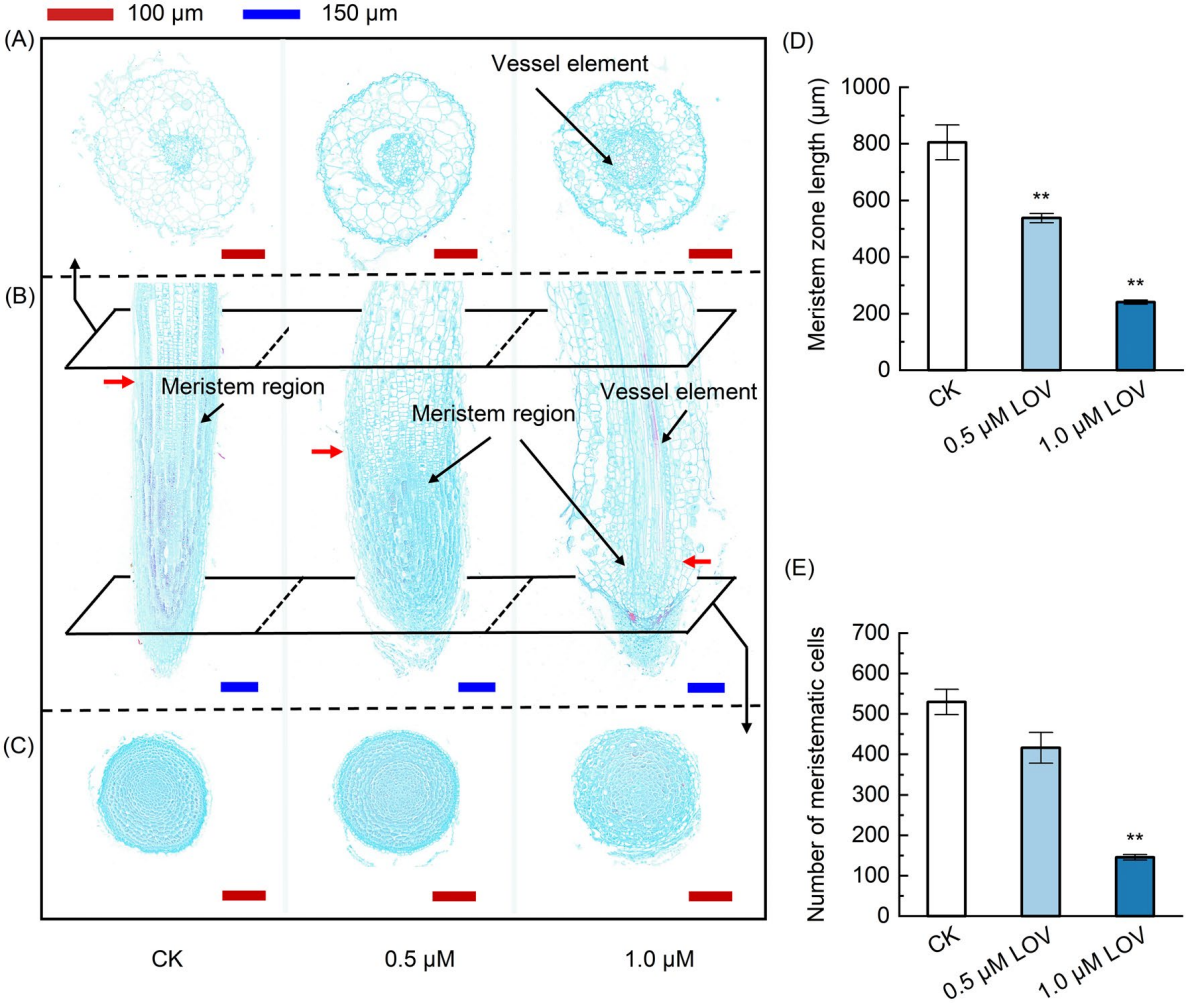
Effect of LOV on the lateral root apical meristem zone of soybean seedlings. Micrographs (**A**) and (**C**) show demonstrate transverse sections, and (**B**) longitudinal sections. Fast green FCF and safranin O were used to stain the sections. The lignified or corkified cell wall and vessel element will be dyed red and other tissues will be dyed green. Red scale bars = 100 μm, Blue scale bars = 150 μm. The red arrowhead indicates the upper boundary of the meristem zone. (**D**) The effect of LOV on the length of the apical meristematic zone and (**E**) the number of meristematic cells. The data represent the mean ± SE of three biological replicates. Symbol * denotes significant difference from CK at *p* < 0.05; ** at *p* < 0.01 by Student’s t-tests.

According to the above obtained result, LOV significantly inhibited the development of lateral roots. Tissue sections of LOV-treated lateral root tips in soybean seedlings were employed to further explore the causes of stagnated lateral root growth. As shown in Fig. 3B, the length of the apical meristematic zone was significantly shorter after LOV treatment. Specifically, the length of the apical meristem zone was reduced by 33.25% and 70.13% after 0.5 and 1.0 μM LOV treatments, respectively (Fig. 3D). The root tip cell became more irregular and loose compared with CK (Fig. 3B,C). More particularly, the meristem cells only existed in a small specific area of the root tip after treating with 1.0 μM LOV, and the number of meristematic cells was only 27.50% of that of CK (Fig. 3C,E). In addition, we found that mature vessel elements were observed at the areas close to the root tip treated with 1.0 µM LOV (Fig. 3A,B). According to the demonstrated results, the phenomenon that LOV inhibited the lateral roots’ development may be caused by the restraining cell division in the meristematic zone.

### Decrease in sterols and soyasapogenol content in soybean seedlings after LOV treatment

**Figure 4:**
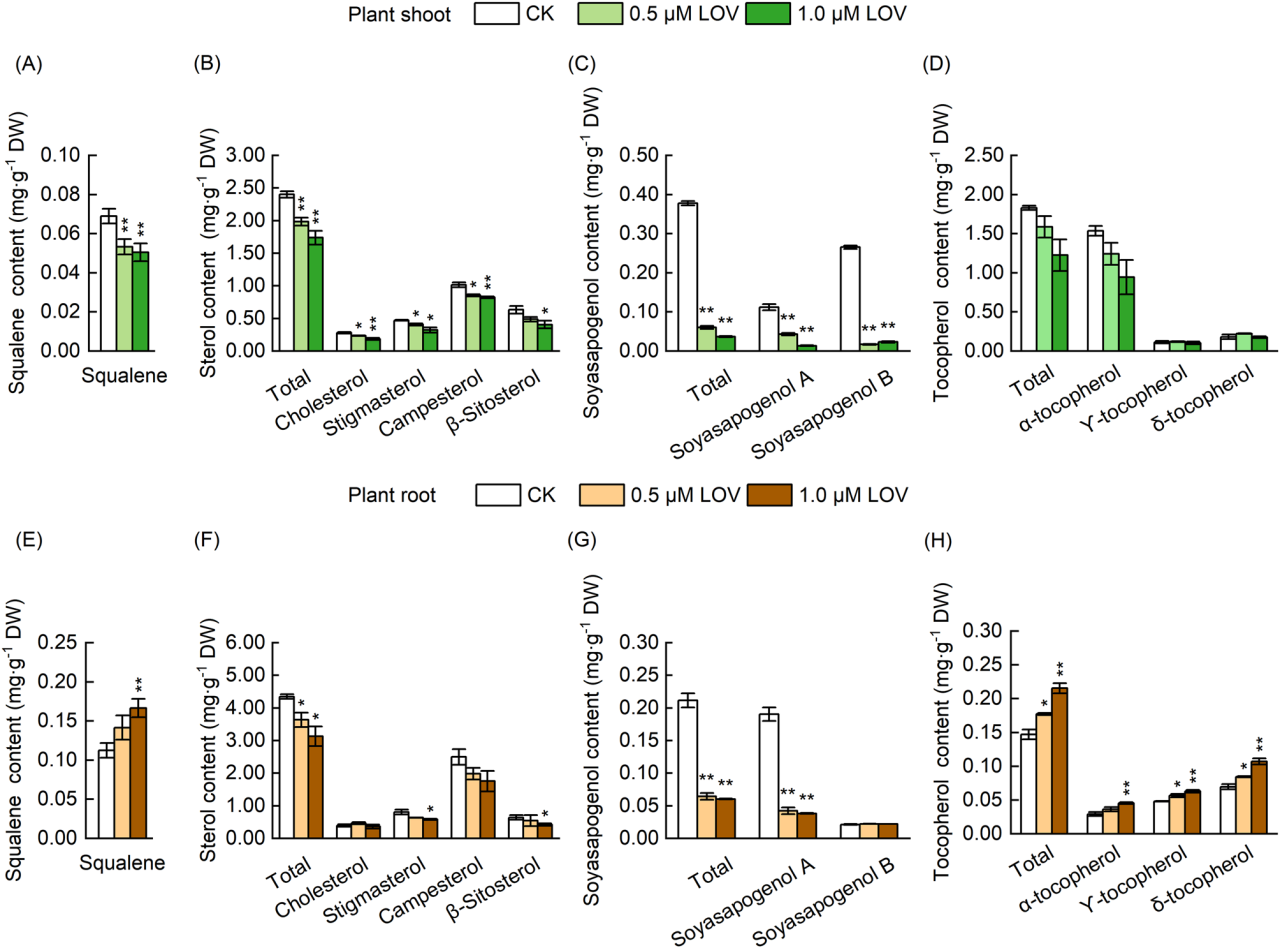
Content changes of isoprenoids in LOV-treated soybean seedlings. (**A**) Content changes of squalene, (**B**) sterols, (**C**) soyasapogenol, and (**D**) tocopherols in plant shoot after LOV treatment. (**E**) Content changes of squalene, (**F**) sterols, (**G**) soyasapogenol, and (**H**) tocopherols in LOV-treated plant root. The data represent the mean ± SE of three biological replicates; the symbol * denotes significant difference from CK at *p* < 0.05; ** at *p* < 0.01 by Student’s t-tests.

In order to clarify the effect of HMGR on the metabolites related to the MVA and MEP pathways in soybean seedlings, the content of several representative substances was determined by HPLC, including squalene, sterols, soyasapogenol, and tocopherols in LOV-treated soybean seedlings. The content of squalene was significantly reduced in the shoot, but higher in the root after LOV treatment (Fig. 4A,E). The dose effect of LOV on soybean seedling development corresponded well with the reduction in sterols after LOV treatment. After 1.0 µM LOV treatment, the content of the total sterol in plant shoot and root was significantly reduced to 72.38% and 72.10% of the CK, respectively (Fig. 4B,F). At the same time, the total soyasapogenol content decreased by 90.25% and 71.48%, respectively (Fig. 4C,G). As the related metabolites of the MEP pathway, the tocopherols (Fig. 4D) and chlorophyll (Supplementary Fig. S3) biosynthesis weren’t significantly affected in plant shoot after LOV treatment. Whereas, the content of the total tocopherol increased significantly in the roots, compared with that in plant shoot (Fig. 4H). These results indicated that LOV effectively inhibited the biosynthesis of the metabolites in the MVA pathway.

### Effects of LOV on transcription levels of genes related to MVA and MEP pathways

**Figure 5:**
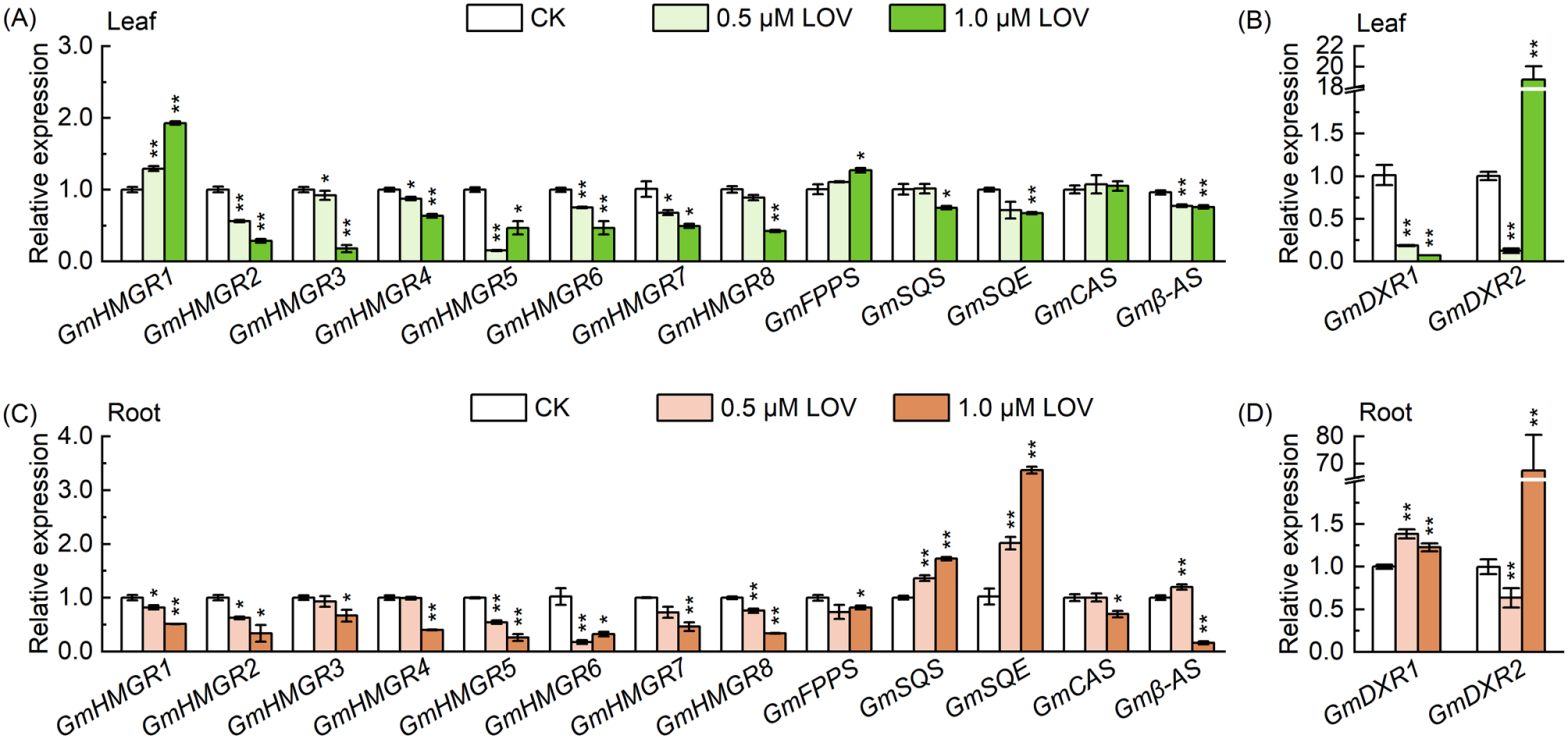
Expression changes of MVA and MEP pathways-related genes in LOV-treated soybean seedlings. (**A**) Transcript levels of HMGR (*GmHMGR1-8*), Farnesyl diphosphate synthase (*GmFPPS*), Squalene synthase(*GmSQS*), Squalene epoxidase (*GmSQE*), Cycloartenol synthase (*GmCAS*), *β*-amyrin synthase (*Gmβ-AS*), 1-Deoxy-D-xylulose 5-phosphate reductoisomerase (*GmDXR*) in soybean leaves; (**C**) and (**D**) Transcript levels in soybean roots. The data represent the mean ± SE of three biological replicates; the symbol * denotes significant difference from CK at *p* < 0.05; ** at *p* < 0.01 by Student’s t-tests.

To investigate whether changes in the metabolites were related to alterations in gene expression before and after LOV treatment, expression changes of selected genes (*GmHMGR1-8* and *GmDXR1-2*) were analysed in the soybean seedling roots and leaves (Fig. 5). A decrease in the relative expression of *GmHMGR2-8* in soybean seedling roots and leaves was observed after LOV treatment, particularly after 1.0 μmol∙L^−1^ LOV treatment (Fig. 5A,C). However, the expression of *GmDXR2* in the leaves and *GmDXR1-2* in the roots were significantly increased after 1.0 μmol∙L^−1^ LOV treatment (Fig. 5B,D). In addition, the gene expression levels of some important intermediate metabolite synthases also changed significantly in the MVA pathway. As is indicated in the results, the transcriptional levels of related genes in the MVA pathway were connected with the content changes of sterols, and soyasapogenol biosynthesis in soybean.

### Subcellular localization of GmHMGR4 and GmHMGR6

**Figure 6:**
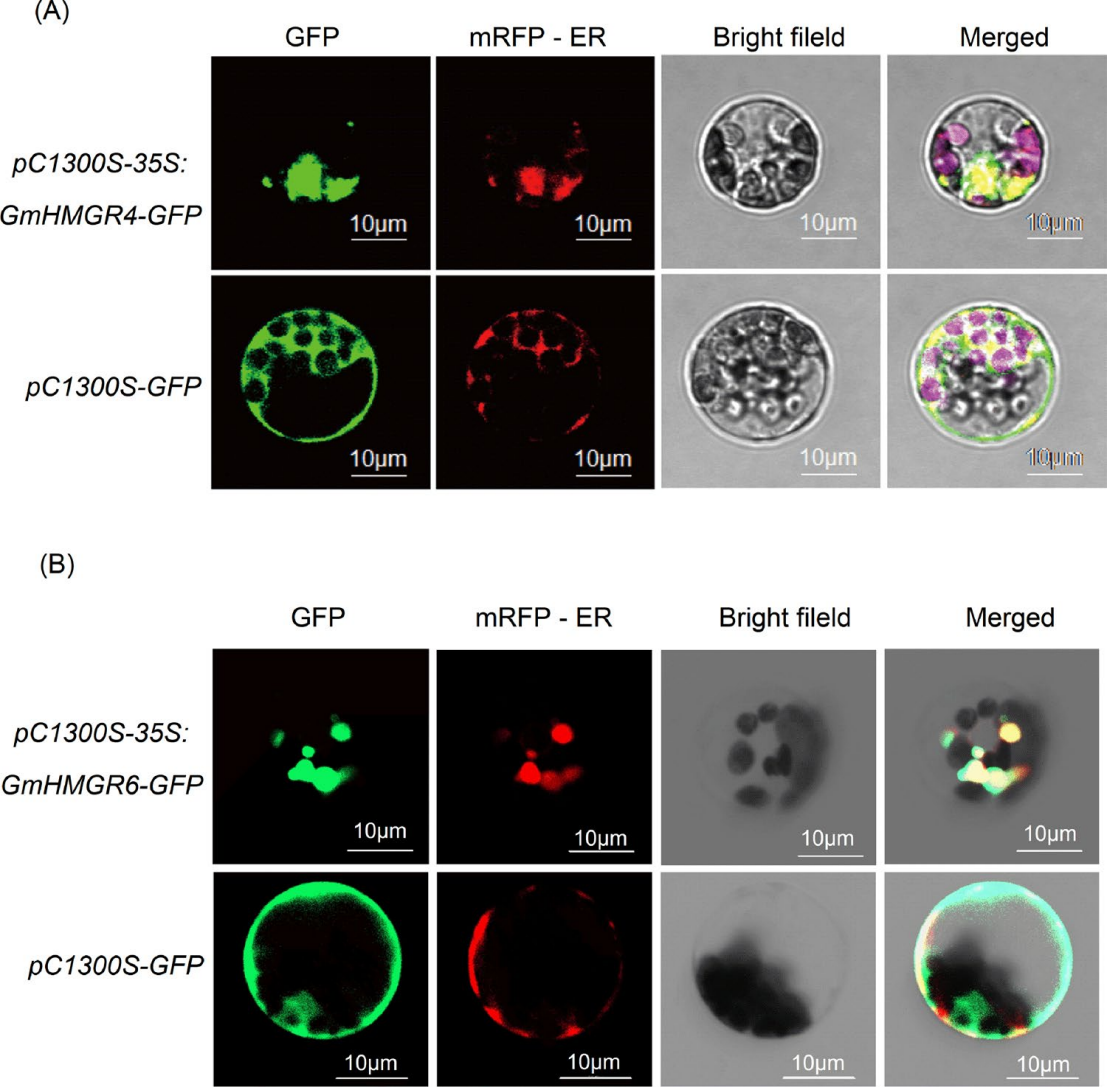
Subcellular localization of (**A**) GmHMGR4 and (**B**) GmHMGR6 proteins in *A. thaliana* protoplasts. Green fluorescence indicates a fusion protein signal for *pC1300S-35S:GmHMGR4-GFP* and *pC1300S-35S:GmHMGR6-GFP*. The red signal indicates mRFP-ER (Endoplasmic reticulum protein marker fused with mRFP) fluorescence. GFP and mRFP-ER fluorescence images were digitally combined to get the merged images.

To determine the sub-cellular localization of GmHMGR4 and GmHMGR6 proteins, the *pC1300S-35S:GmHMGR4-GFP* and *pC1300S-35S:GmHMGR6-GFP* fusion expression vectors and the *pC1300S-ER-mCherry-HDEL* vector were transformed into *A. thaliana* mesophyll protoplasts, respectively. *A. thaliana* protoplasts that were transfected with GmHMGR4-GFP, GmHMGR6-GFP fusion proteins have a kind of endoplasmic reticulum (ER) mass in the same position with mRFP-ER (endoplasmic reticulum marker) respectively, but do not have a typical ER network (Fig. 6A,B). According to related researches, HMGR proteins are located in the ER^35–37^. The membrane structural domains of plant HMGR can induce ER proliferation and promote the biogenesis of organized smooth endoplasmic reticulum (OSER) structure that are highly dynamic entities^35,36,38–40^. To better define subcellular location the GmHMGR proteins, further experiments on transforming GmHMGR into *A. thaliana* or *Nicotiana benthamiana* leaves are needed to conduct.

### Effects of overexpressing *GmHMGR4* and *GmHMGR6 *on root growth in *A. thaliana*

**Figure 7:**
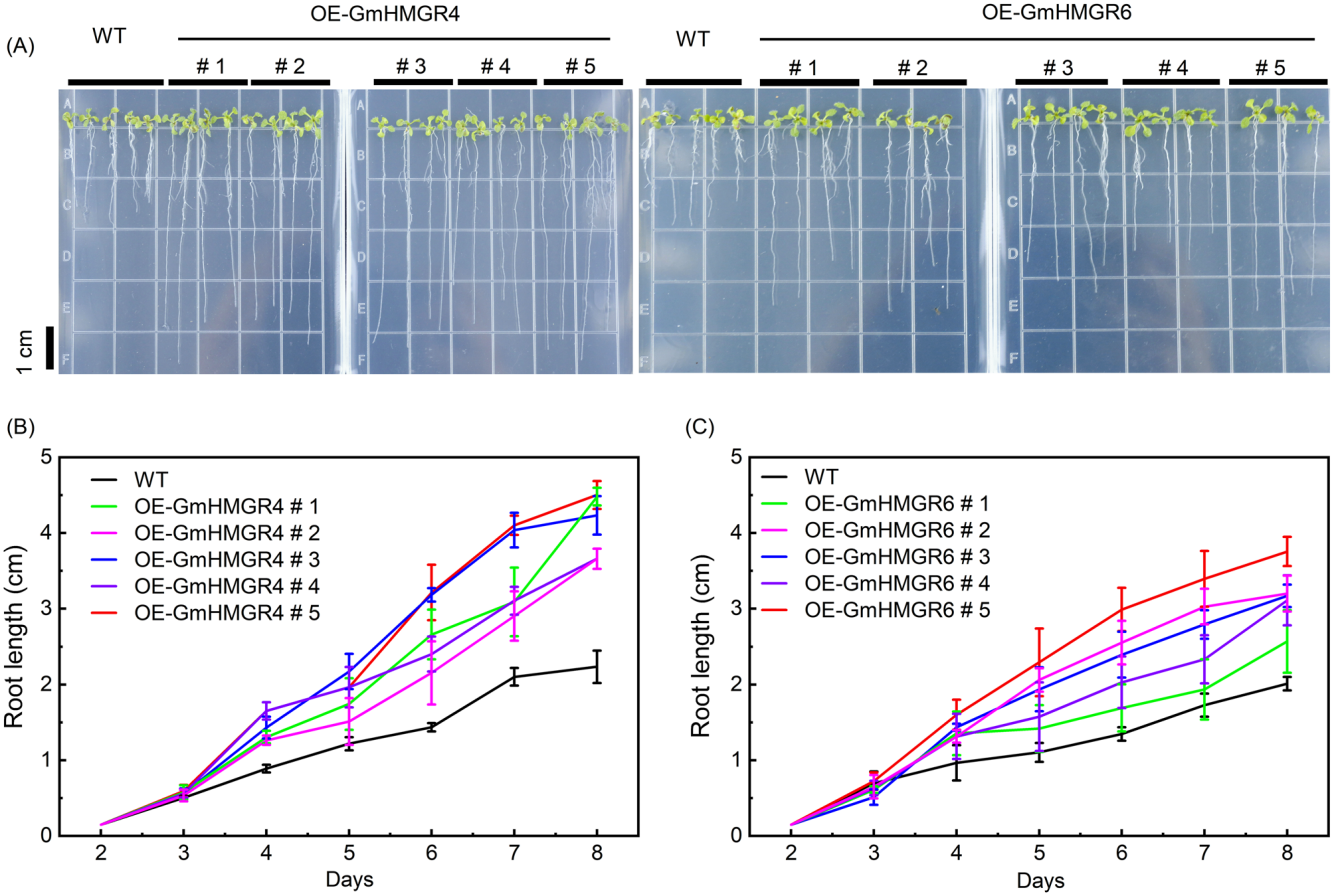
*A. thaliana* primary root growth during an 8-day period for the WT, OE-GmHMGR4s and OE-GmHMGR6s. (**A**) Growth phenotypes of *A. thaliana* grown for 8 days in a vertically placed MS medium. (**B**) and (**C**) Changes in root length between WT and transgenic *A. thaliana*. WT: wild type, n ≥ 15 roots per overexpression strain.

Previous studies have demonstrated that *GmHMGR4* and *GmHMGR6*, which are highly expressed during soybean growth and seed maturation, play an important role in the synthesis of soybean isoprenoid^16,33^. In addition, *GmHMGR4* and *GmHMGR6* encode typical HMGR enzymes. To further investigate the effect of *GmHMGR* during plant growth, we transformed the *GmHMGR4* and *GmHMGR6* genes into *A. thaliana* respectively, and the root length of *A. thaliana* OE-GmHMGE4s and OE-GmHMGE6s was measured. The length of the primary root was significantly increased both in OE-GmHMGR4s and OE-GmHMGR6s compared with the wild type (WT) from 3 to 8 days old seedlings (Fig. 7A). The average primary root length in OE-GmHMGR4s and OE-GmHMR6s were 83.96% and 57.21% higher than that in the WT for 8-day-old seedlings (Fig. 7B,C). In addition, the HMGR enzyme activity in transgenic *A.thaliana* was significantly higher than that of WT except for the OE-GmHMGR4 # 1 strain (Supplementary Fig. S4). The above results demonstrated that the overexpression of *GmHMGR4* and *GmHMGR6* may promote root growth in *A. thaliana.*

### Effects of overexpressing *GmHMGR4* and *GmHMGR6 *on isoprenoid metabolism in *A. thaliana*

**Figure 8:**
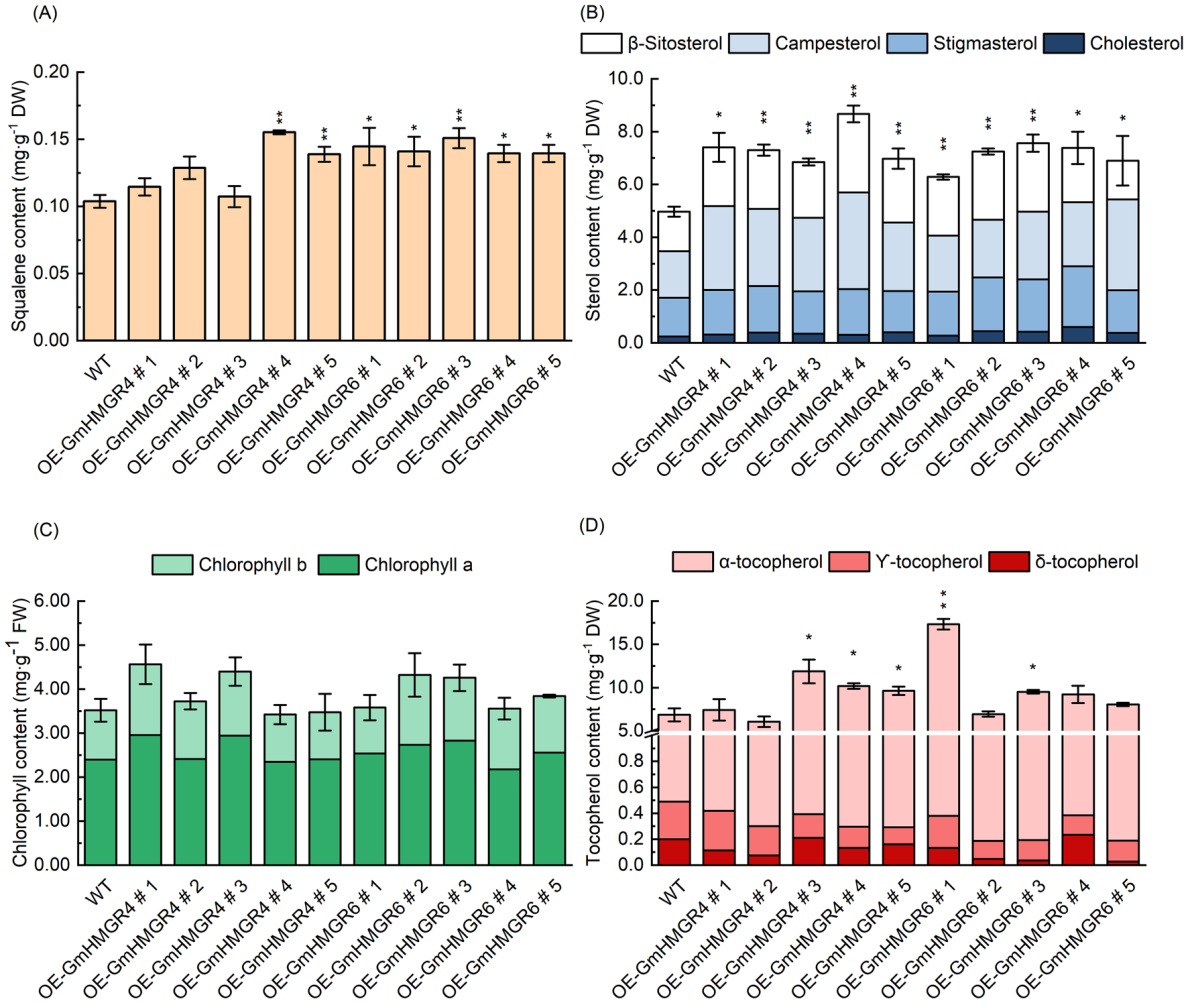
Content changes of squalene, sterols, chlorophyll, and tocopherol in overexpressed *A. thaliana* lines. (**A**) HPLC analysis of squalene content (mg·g^−1^, DW) in *A. thaliana*. (**B**) HPLC analysis of sterol content (mg∙g^−1^, DW) in *A. thaliana*, different colors represent β-sitosterol, campesterol, stigmasterol, and cholesterol. (**C**) Chlorophyll a and b content in leaves of *A. thaliana* (mg∙g^−1^, FW). (**D)** HPLC analysis of tocopherol content (mg∙g^−1^ DW), different colors represent α-tocopherol, ϒ-tocopherol, and δ-tocopherol. The data represent the mean ± SE of three biological replicates; the symbol * denotes significant difference from wild type (WT) at *p* < 0.05; ** at *p* < 0.01 by Student’s t-tests.

OE-GmHMGR4s and OE-GmHMGR6s were compared with WT to investigate the content changes of squalene, sterols, chlorophyll, and tocopherols. The content of sterols and squalene was significantly increased (Fig. 8A,B). OE-GmHMGR4s exhibited greater sterols increases (37.84–74.58%) than OE-GmHMGR6s (26.45–52.30%). Changes in sterols levels of the OE-GmHMGR4s and OE-GmHMGR6s coincided with alterations in primary root growth (Fig. 7). Interestingly, tocopherol content, one of the MEP pathway products, was also increased in comparison with the WT (Fig. 8D). While chlorophyll contents did not observe significant differences in OE-GmHMGR4s and OE-GmHMGR6s lines compared with the WT (Fig. 8C). The obtained results clearly suggested that *GmHMGR4* and *GmHMGR6* affected isoprenoid metabolism in *A. thaliana.*

## Discussion

In the MVA pathway, HMGR is a vital rate-limiting enzyme^6,7,41^. Compared with the single HMGR found in animals, HMGR in archaea, eubacteria, and plants is encoded by a multigene family whose various isoforms exhibit diverse spatial and temporal gene expression patterns^7^. GmHMGR was encoded by a family of eight genes in soybean. So, knowing their function is important for soybean development and isoprenoid metabolism. However, it is difficult to study the function of *GmHMGR1-8* by knocking out or overexpressing *GmHMGR1-8* in soybean respectively. The LOV’s 3,5-dihydroxyheptanoic acid structure is remarkably similar to that of HMG-CoA, which enables LOV to competitively inhibit the binding of HMG-CoA to the active site of the HMGR enzyme. Thus, LOV was generally used to study the function of HMGR^29,31,32,42,43^. A recent study solved the structures of *A. thaliana* HMGR in *apo* form and in complex with a statin, which provides new proof to the finding that LOV could be used to study the function of HMGR in plants^30^. To investigate whether LOV interacts with GmHMGR 1–8 proteins, we analysed the binding energies from the interactions of GmHMGR proteins’ docking sites with LOV and HMG-CoA via AutoDockVina. GmHMGR1-GmHMGR8’s binding energies to LOV were all lower than their binding energies to HMG-CoA. Although GmHMGR2 lacks HMG-CoA binding motif, LOV can still establish a stable conformation with NADP(H) binding motif II. Like other GmHMGR proteins, the binding energy of LOV to GmHMGR2 (− 6.2 kcal∙mol^−1^) is still lower than that of HMG-CoA to GmHMGR2 (− 5.7 kcal∙mol^−1^). In our study, the relative expression of *GmHMGR1-8* and HMGR activity in the roots of soybean seedlings were down-regulated after LOV treatment. The evidence suggested that employing LOV to explore the function of *GmHMGR* was feasible. Herein, we studied the function of *GmHMGR* in soybean growth and development, especially the establishment of primary roots, by using LOV in soybean. In addition, *GmHMGR4* and *GmHMGR6* were overexpressed in *A. thaliana* to verify their effects. However, to better understand the role of *GmHMGR* genes, it could be preferable to overexpress *GmHMGR* genes in soybean.

**Figure 9:**
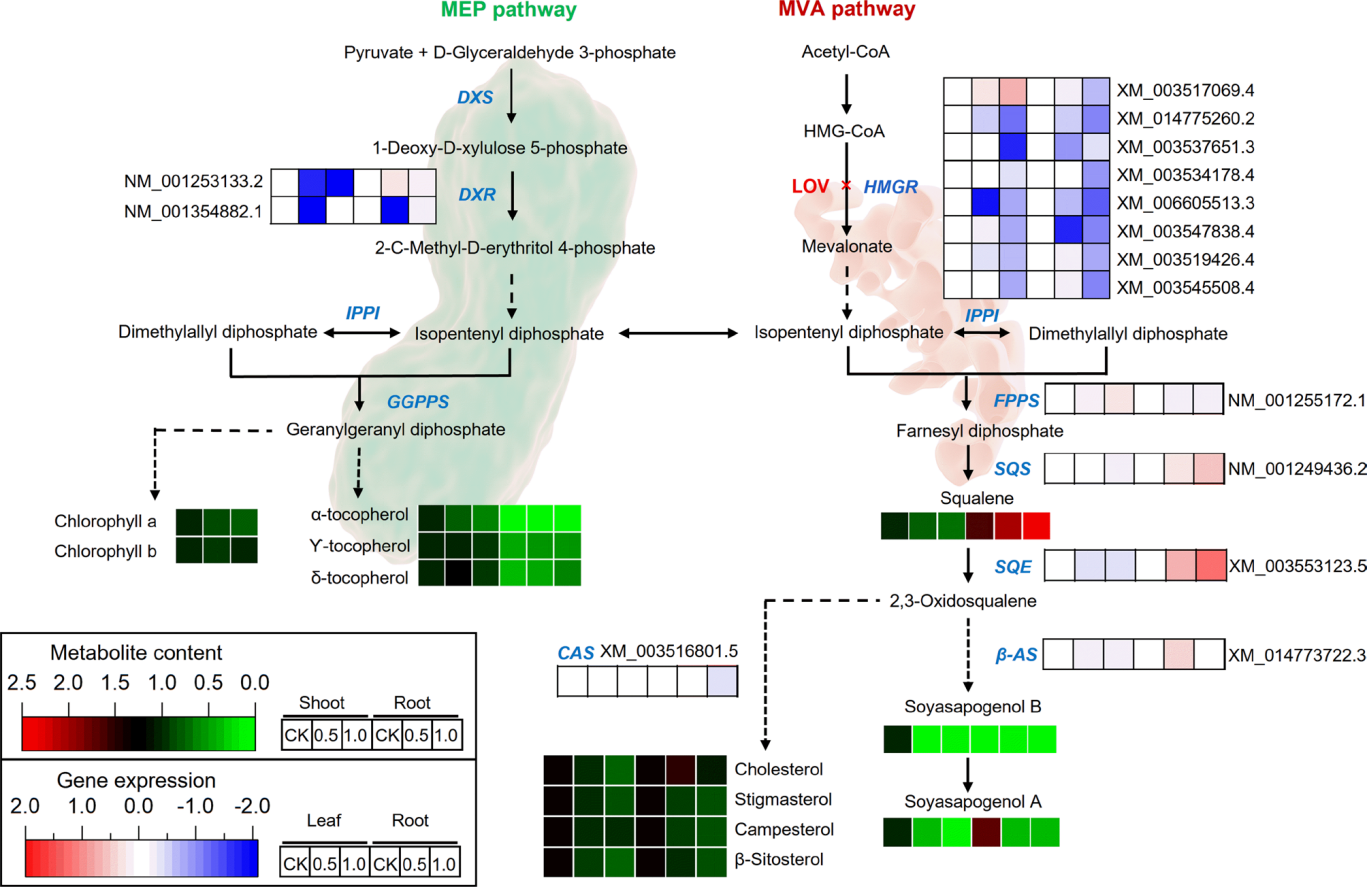
Inhibition of HMGR alters gene expression levels and isoprenoid biosynthesis in the MVA and MEP pathways of soybean. Some important isoprene compounds were indicated in black fonts, and the key enzyme genes were indicated in blue italic fonts. Red and green scales show the change in isoprenoids content compared with CK, the data was calculated based on the isoprenoids content of the shoot. Upregulated isoprenoids were marked in red, and reduced isoprenoids were displayed in green. A light red and blue color scale depict gene expression in comparison to CK. Upregulated genes are shown in light red, and downregulated genes are shown in blue. Enzyme abbreviations: DXS: 1-Deoxy-d-xylulose 5-phosphate synthase; DXR: 1-Deoxy-d-xylulose 5-phosphate reductoisomerase; IPPI: isopentenyl diphosphate ∆-isomerase; GGPPS: geranylgeranyl diphosphate synthase; FPPS: farnesyl diphosphate synthase; SQS: squalene synthase; SQE: squalene epoxidase; CAS: cycloartenol synthase; *β-*AS: *β*-amyrin synthase.

To further explore the mechanism of GmHMGR affecting growth and primary root development in soybean, the changes in phenotype, transcript, and metabolite were analyzed after LOV treatment in soybean and overexpression of *GmHMGR4* and *GmHMGR6* in *A. thaliana*. Previous researches have showed that HMGR plays a critical role in controlling the flow of carbon within the MVA pathway and affects isoprenoid metabolism and plant growth^44,45^. According to the previous studies, LOV and F-244 (selective inhibitors of HMGS in the MVA pathway) inhibited root development in plants^31,46^. LOV significantly inhibited the root growth of soybean seedlings, as well as the sterol biosynthesis and HMGR enzyme activity. *A. thaliana* overexpression lines showed higher HMGR enzyme activity, squalene and sterol content. In addition, the development of the primary root being faster than that of the WT in *A. thaliana* overexpression lines, which was consistent with the overexpression of the *HMGR* gene in other plants, such as *S. tuberosum*^47^, *P. trichocarpa*^48^. To date, mutant *SMT* genes in the MVA pathway caused reduced β-sitosterol and stigmasterol content and delayed root development^49,50^. Additionally, the defects in lateral root development resulting from mutant *SMT2* and *SMT3* can be recovered by exogenous supplementation of β-sitosterol^51^. These results suggested that the decrease in stigmasterol and β-sitosterol content appeared to be the main factor responsible for the developmental defects of soybean seedlings. Many studies have reported that the mutant seedlings exhibited cell elongation defects, abnormal cell morphologies and severely disordered cell rows in *A. thaliana* sterols synthesis-deficient mutation^49,52,53^. Similar to the previous results, we found that the length of the meristematic zone and the number of meristematic cells were significantly reduced in the lateral roots of soybean seedlings treated with LOV, and that the cell morphology was disordered in the root tips treated with 1.0 μM LOV. Furthermore, LOV-treated soybean seedlings had considerably lower total sterol content than the control. For individual sterol, stigmasterol and β-sitosterol were also significantly reduced. That is, reduced sterols content may cause abnormal root cell development, which may lead to the repressed phenotype of roots. So we hypothesized that *GmHMGR* affected the root development for several reasons: down-regulation of some isoprenoid-related genes (*GmHMGR* and *GmCAS* in root) and HMGR activity, a significant reduction in sterols content and a reduction in the root apical meristem (Fig. 9).

To investigate whether the changes of isoprenoid metabolism in soybean seedlings after LOV treatment were linked to the expression of genes, we analyzed the expression of related genes in the MVA and MEP pathways. The results of RT-qPCR indicated that LOV caused the expression changes of *GmHMGR* and those of some genes in the MEP pathway, thereby affecting downstream intermediates content. The transcript level of *Gmβ-AS* gene was significantly decreased after 1.0 μM LOV treatment. Correspondingly, the content of soyasapogenol, a representative product of soybean triterpenoids, was significantly reduced after LOV treatment. However, the LOV treatment increased the squalene content in the roots while decreasing it in the shoot. The changes in squalene content were consistent with changes in *GmSQS* gene expression in roots versus shoots. Furthermore, the expression pattern of *GmHMGR1* was different from that of other *GmHMGR* genes. According to a previous study, the HMGR was subjected to strict feedback control through multiple mechanisms to ensure cells constantly produced essential isoprenoids^54–56^. We speculate that the up-regulated expression of *GmHMGR1* was mediated by a feedback mechanism, and that *GmHMGR1* may play a special role in soybean growth and development. However, the reasons behind *GmHMGR1*’s distinct expression pattern compared to other *GmHMGR* genes must be investigated further. Inconsistent with the significant reduction in sterols content, the expression of the *GmCAS* gene in plant shoot was not significantly inhibited. The lack of correlation between these gene expression patterns and the accumulation of isoprenoid metabolites suggested that post-transcriptional regulation processes may play an important role in regulating flux through the isoprenoid metabolic pathway^29^. According to earlier researches, LOV had a very specific impact on the mitogen-activated protein kinase (MAPK) pathway and could modulate cellular signal transduction independently of the MVA pathway in vascular smooth muscle cells^57^. However, whether LOV affects signal transduction pathways in plants needs to be investigated. LOV also affected the cytokinin synthesis pathway^58,59^, and the branched-chain amino acids (BCAAs) biosynthesis pathway in plants^60^. In addition, recent studies have shown that a new enzymatic mechanism for the biosynthesis of triterpenes may exist in plants^61^. It is necessary to conduct more research into the impacts of LOV on soybean since the regulation of the isoprenoid content in soybean may be complicated.

The MVA and MEP pathways take place in different subcellular compartments in plants. Under normal growth conditions, the inhibitor block of the MVA pathway could not be rescued by the activity of the MEP pathway and vice versa^6^. Wang et al*.*^5^ found that overexpression of *BjHMGS* genes in *A. thaliana* did not significantly affect gene expression in the MEP pathway or the final products’ content. Whereas some studies had found molecular and metabolic interactions between the MVA and MEP pathways^7,17,31^. According to Liao et al.^12^, overexpression of the *HMGS* gene of mustard increased α-tocopherol and carotenoid levels in transgenic tomatoes. By overexpressing the *PtHMGR* gene enhanced the *GPS* and *GPPS* expression in *Populus euramericana*, resulting in stimulating the crosstalk between the MVA and MEP pathways^23^. Likewise, our results indicate that inhibited *GmHMGR* of the MVA pathway in soybean significantly affected the tocopherols’ content in the MEP pathway, whereas chlorophyll was not significantly changed. That is, there may exist crosstalk between the MVA pathway and MEP pathway in soybean.

## Materials and methods

### Soybean materials and inhibitor treatment

Soybean (*Glycine max* (L.) Merr.) cv. ‘Wuhei’ (provided by Soybean Germplasm Innovation and Utilization Laboratory, Shanxi Agricultural University) was used in this experiment. The materials used adhere to the relevant institutional, national, and international guidelines and legislation. LOV treatment was performed as previously reported^3^. We dissolved 10 mM fresh LOV (Solarbio Science, China) in hydrolysate (0.2% NaOH and 20% ethanol, w/v) and incubated the corresponding hydrolysate at 65 °C for 1.5 h to hydrolyze the lactone ring. After that, it was stored at 4 °C after being filter sterilized with a filter membrane (0.22 μm). During the experiment, LOV stock solutions were added into MS medium and diluted to create the final working solution concentrations of 0.5 and 1.0 µM. The control samples were merely treated with water distillate. ‘Wuhei’ seeds were sterilized and germinated in the dark at 25 °C. Germinating seeds with similar growth sizes were moved to MS medium having different LOV concentrations and grew at 25 ℃ for 16-h-light/8-h-dark cycle conditions. Each bottle planted 3 soybean seedlings. For each treatment, three replicates of 20 bottles (60 plantlets) were conducted. After 25 days, the soybean seedlings were harvested for phenotype observation, including plant height, plant weight (fresh weight, FW), root–shoot ratio, primary root length, root weight (FW), number of lateral roots, and lateral root length distribution. Then, some of the seedlings from each treatment were taken to make tissue sections. The remaining seedlings were divided into three groups. Fresh leaves from the first group were taken for determination of chlorophyll content. The second group was dried in a freeze dryer for 48 h to a constant weight for analyzing the content of isoprenoids, including squalene, sterols, soyasapogenol and tocopherols. We froze the last group in liquid nitrogen and kept it at − 80 ℃ to study the related genes’ expression.

### Sequence analysis of GmHMGR proteins and molecular docking verification

The cDNA sequences of *GmHMGR1-GmHMGR8* were derived from the NCBI database (NCBI reference sequences: XM_003517069.4, XM_014775260.2, XM_003537651.3, XM_003534178.4, XM_006605513.3, XM_003547838.4, XM_003519426.4 and XM_003545508.4). The amino acid sequences of GmHMGR1-GmHMGR8 were aligned by the DNAMAN program. Conserved domains of GmHMGR were analyzed in MEME (http://memesuite.org). Homologous modeling was performed in Phrye2 (http://www.sbg.bio.ic.ac.uk/phyre2/html/page.cgi?id=index), and the structures with the highest scores were selected for molecular docking GmHMGR with HMG-CoA and LOV separately. The data files of HMG-CoA and LOV were obtained from Protein Data Bank (https://www.rcsb.org/). HMG-CoA, LOV and GmHMGR were modified by AutoDockTools 1.57, including ligand extraction, hydrogen addition, and water removal. By docking using AutoDockVina, the conformations with the lowest docking binding energy were chosen as the evaluation criterion.

### Microscopic section of soybean lateral roots

The lateral roots were fixed in formalin–acetic acid–methanol (FAA) according to the method of Livingston et al*.*^62^. Blocks were sectioned with a Leica RM2016 rotary microtome at 5 μm. Fast green FCF and safranin O were used to stain the sections. The lignified or corkified cell wall and vessel element will be dyed red and other tissues will be dyed green. Images were captured with a Nikon Eclipse E100. Subsequently, the length of the meristematic zone and the number of meristematic cells were analyzed using ImageJ 1.53.

### Transient expression of recombinant proteins (GmHMGR4-GFP and GmHMGR6-GFP) on *A. thaliana* protoplasts

*GmHMGR4* and *GmHMGR6* cDNA sequences were cloned into a pC1300s vector with the CaMV 35S promoter and eGFP (enhanced green fluorescent protein). The *pC1300S-35S:GmHMGR4-GFP* and *pC1300S-E.R-mCherry-HDEL* (plant endoplasmic reticulum markers) were transfected into *A. thaliana* mesophyll protoplasts, *pC1300S-35S:GmHMGR6-GFP* and *pC1300S-E.R-mCherry-HDEL* also go through the same transfection. On the second day following transfection, confocal laser scanning microscopy (OLYMPUS FV 1200) was used to detect the fluorescence from fusion proteins and organelle markers.

### Transformation of *A. thaliana*

The *GmHMGR4* and *GmHMGR6* cDNA sequences were cloned into the expression vector pC3300s directed by the CaMV 35S promoter, respectively. Briefly, we used primers with *Sac*I and *Bam*HI sites (underlined *GmHMGR4*, 5′-CTCTCGAGCTTTCGCGAGCTCCCCATTTCCCTTCCAATCT-3′ and 5′-CTGCAGGTCGACTCTAGAGGATCCCCCCACCATCATCAATACCA-3′; *GmHMGR6*, 5′-CTCTCGAGCTTTCGCGAGCTCAAACAAGGGTTGCACGCTCT-3′ and 5′-CTGCAGGTCGACTCTAGAGGATCCACCCCCTC CCACCATCAAT-3′) to amplify the cDNA of *GmHMGR4* and *GmHMGR6*. The above obtained PCR products were enzyme-digested and cloned into the vector pC3300s (named *pC3300S-35S:GmHMGR4* and *pC3300S-35S:GmHMGR6*)*.* The *pC3300S-35S:GmHMGR4* and *pC3300S-35S:GmHMGR6* were transformed into *A. thaliana* (Col-0, provided by Wuhan Towin Biotechnology Company Limited, China) using *Agrobacterium* GV3101. T_0_ transformants *A. thaliana* seeds were selected by MS containing glufosinate ammonium (100 mg∙mL^−1^). Then the corresponding insertions of transgenes were verified by PCR using the bar gene primer (Supplementary Fig. S5). After vernalizing at 4 ℃ for 24 h, the transgenic lines of *A. thaliana* grew in MS medium at 21℃ under 16-h-light/8-h-dark conditions. T_3_ transgenic homozygous lines were utilized to detect isoprenoid’s content and observe root growth. The chlorophyll content was analyzed by using fresh rosette leaves of 30-day-old *A. thaliana.* Other plants in this experiment were freeze-dried to analyze the content of squalene, sterols and tocopherols. Additionally, seeds were planted in square plates with MS and 1% agar for root growth tests, and the main root’s average length was measured daily.

### Measurement of chlorophyll

As described by Simpson et al*.*^63^, the total chlorophylls were extracted from 0.2 g fresh sample using 8 mL ethanol/acetone/hexane (1:1:2, v/v). Absorbance was measured at 470, 645, and 663 nm respectively. The amount of chlorophyll a and b was calculated by the formulae from Zhang et al*.*^64^.

### HPLC analysis of soyasapogenol from soybean

The extraction method of soyasapogenol A and B was performed according to Rupasinghe et al*.*^65^. The 0.4 g sample was extracted by 80% ethanol solution containing 0.01% acetic acid at 25 °C for 24 h and then hydrolyzed with 1 M HCl-methanol for 200 min at 75 °C. The content of soyasapogenol was detected by HPLC (Agilent 1260 Infinity equipped with DAD). An isocratic elution was composed of 60% acetonitrile and 0.1% acetic acid. As previously described, the solvent flow rate was set at 1.0 mL∙min^−1^ and UV absorption was monitored at 205 nm.

### Isoprenoids composition analysis

The analysis of squalene and sterols (including cholesterol, stigmasterol, campesterol, and β-sitosterol) was based on Slavin et al.^66^. 0.2 g freeze-dried sample was extracted and saponified. The contents of sterols and squalene were analyzed by HPLC using 100% methanol mobile phases. As previously described, the UV absorption was monitored at 204 nm and the solvent flow rate was set at 1.0 mL∙min^−1^. Sui et al.’s^67^ extraction method was used to extract the tocopherols (α-tocopherol, ϒ-tocopherol, and δ-tocopherol). Specifically, 0.1 g lyophilized sample and 0.125 g ascorbic acid were extracted with ethanol and n-hexane, and dried using a stream of nitrogen at room temperature. The extract was redissolved in methanol for HPLC detection. As described, 100% methanol was used as the mobile phase, whose flow rate was 1.0 mL∙min^−1^. UV absorption was monitored at 295 nm.

### Analysis of HMGR enzyme activity

Soybean saplings' plant shoots and roots as well as the 30-day-old fresh rosette leaves of *A. thaliana* were chosen to measure HMGR enzyme activity. The 0.1 g samples were collected and used for enzyme assays according to the protocol described by the Plant HMG-CoA reductase (HMGR) ELISA Kit (Wuhan Chundu Biotechnology, China).

### Reverse transcription-quantitative PCR (RT-qPCR) analysis

The EZ-10 Total RNA Mini-Preps Kit (Sangon Biotech, China) was used to extract the total RNA. The first strand of cDNA was synthesized from 500 ng total RNA using One-Step gDNA Removal and cDNA Synthesis SuperMix (TransGen Biotech, China). The RT-qPCR reactions were conducted by a Bio-Rad CFX96 system (Bio-Rad, USA). According to Tip Green qPCR SuperMix (TransGen Biotech, China), the RT-qPCR was conducted using 250 ng of cDNA in a 20 µL reaction volume. The two-step amplification program was done at 94 °C for 30 s, 45 cycles at 94 °C for 5 s and 60 °C for 30 s. After 45 cycles, melting curves were drawn heating from 65 to 90 °C with a ramp speed at 0.5 °C∙min^−1^. *GmACTIN* (XM_003552652) was used as the internal housekeeping gene. Three experimental replicates for each reaction were carried out. The quantification of gene expression was calculated by the 2^−ΔΔCt^ method. The gene-specific primers used for the RT-qPCR were presented in Supplementary Table S1.

### Statistical analysis

GraphPad Prism 8.02 was used to perform all statistical analyses. The mean ± standard error for data (n ≥ 3) is used to present the values. *P* values were calculated using Student's t-tests. *P* < 0.05 was regarded as statistically significant.

## Supplementary Information

Supplementary Information.

## Data Availability

The datasets generated during and/or analysed during the current study are available from the corresponding author on reasonable request.
