# Supplementary material for: 3-Hydroxy-3-methylglutaryl coenzyme A reductase genes from Glycine max regulate plant growth and isoprenoid biosynthesis
Source: Sci Rep. 2023 Mar 8;13:3902. doi: 10.1038/s41598-023-30797-4 (PMC9995466; doi:10.1038/s41598-023-30797-4)
Supplement: Supplementary file 1 — Supplementary Information. [file 41598_2023_30797_MOESM1_ESM.pdf]

3-hydroxy-3-methylglutaryl coenzyme A reductase genes from *Glycine max* regulate plant growth and isoprenoid biosynthesis

Shuai Wang[1], Yumei Feng[2], Yin Lou[1], Jingping Niu[3], Congcong Yin[4], Jinzhong Zhao[4], Weijun Du[1*], Aiqin Yue[1*]

[1]College of Agronomy, Shanxi Agricultural University, Taigu, Shanxi, 030801, China

[2]College of Forestry, Shanxi Agricultural University, Taigu, Shanxi, 030801, China

[3]College of Life Sciences, Shanxi Agricultural University, Taigu, Shanxi, 030801, China

[4]Department of Basic Sciences, Shanxi Agricultural University, Taigu, Shanxi, 030801, China

*** Correspondence:**
Aiqin Yue: yueaiqinnd@126.com
Weijun Du: duweijun68@126.com

Supplementary Material

1 Supplementary Figures and Tables

1.1 Supplementary Figures

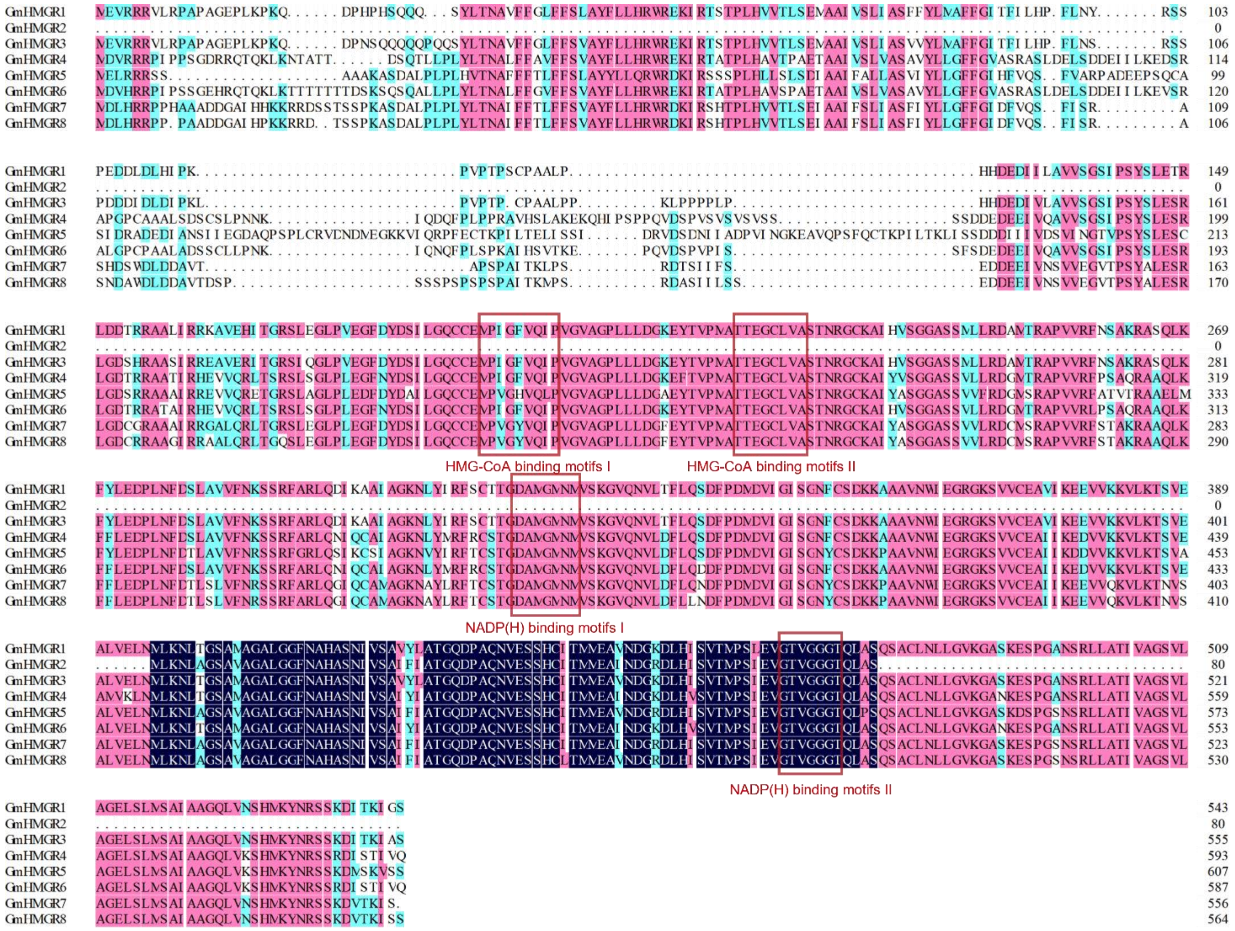

Supplementary Figures S1. Alignment of deduced amino acid sequences of GmHMGR proteins. Dark blue: identity = 100%; red: 75% ≤ identity < 100%; light blue: 50% ≤ identity < 75%; Four conserved active sites of HMGRs, including the two HMG-CoA binding motifs (EMP(I/V)G(Y/F)VQIP and TTEGCLVA) and two NADP(H) binding motifs (DAMGMNM and GTVGGGT), are highlighted in red square box.

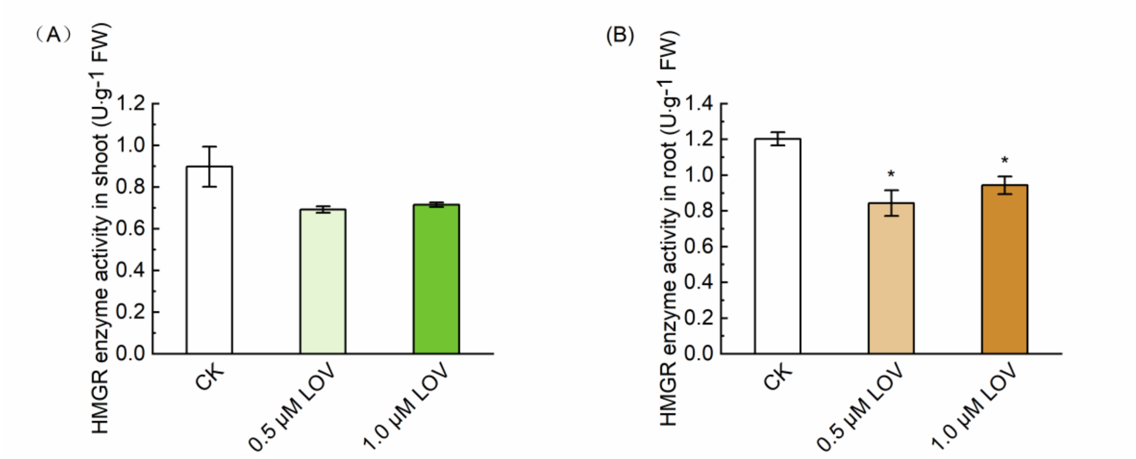

Supplementary Figures S2. Analysis of HMGR enzyme activity in soybean seedlings. (**A**) Plant root. (**B**) Plant root. The data represent the mean ± SE of three biological replicates; the symbol * denotes significant difference from CK at $p < 0.05$; ** at $p < 0.01$ by Student's t-tests.

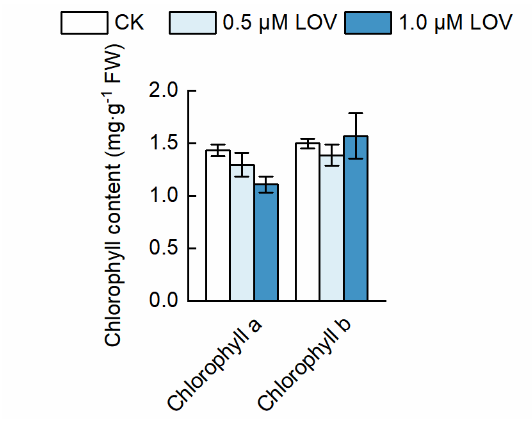

Supplementary Figures S3. Effect of LOV on chlorophyll content in leaves of soybean seedlings. The data represent the mean ± SE of three biological replicates.

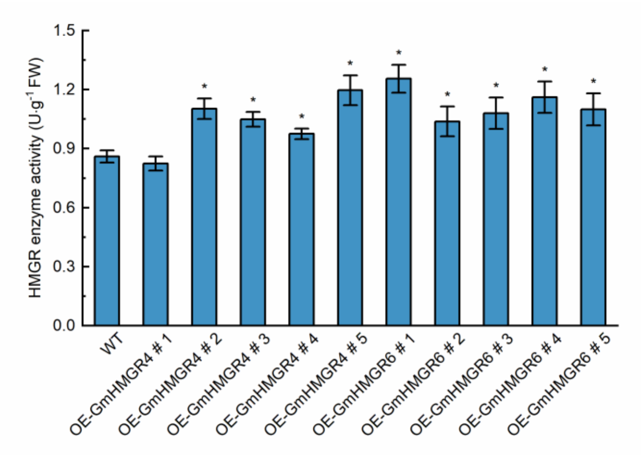

Supplementary Figures S4. Analysis of HMGR enzyme activity in transgenic *A. thaliana*. The data represent the mean ± SE of three biological replicates; the symbol * denotes significant difference from CK at $p < 0.05$; ** at $p < 0.01$ by Student's t-tests.

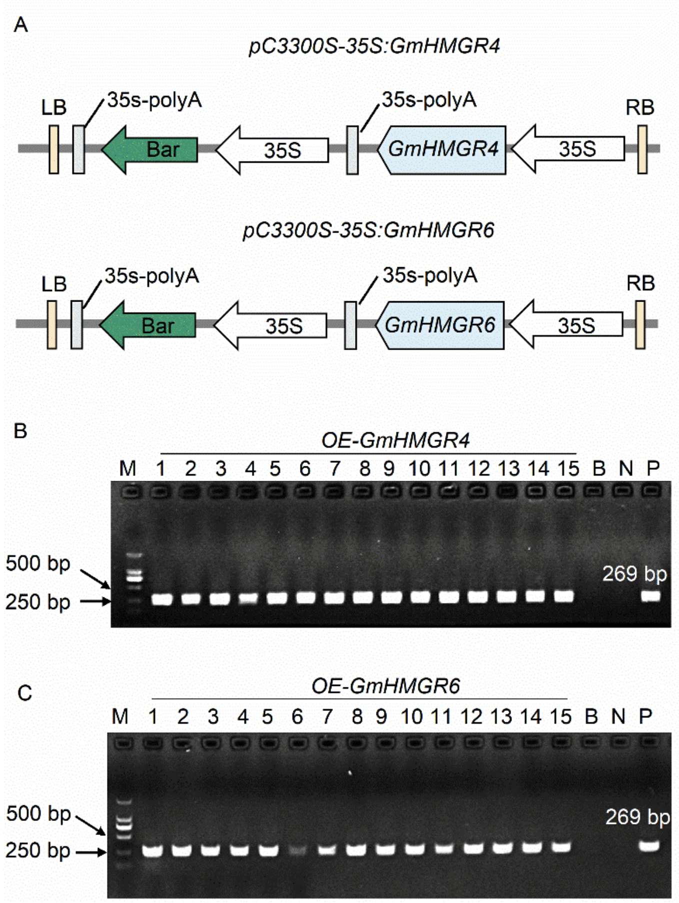

Supplementary Figures S5. PCR analysis of representative transgenic *A. thaliana* HMGR-OEs. (**A**) Schematic map of transformation vector *pC3300S-35S:GmHMGR4* and *pC3300S-35S:GmHMGR6.* Bar: phosphinothricin acetyltransferase gene; 35S: Cauliflower Mosaic Virus 35S promoter; 35s-polyA: cauliflower mosaic virus polyadenylation signal; RB: right border of T-DNA; LB: left border of T-DNA. (**B**) and (**C**) PCR detection of transformed plants by bar forward and bar reverse primer (lane: 1-15 269 bp). Putative *Arabidopsis* HMGR-OEs were designated as *OE-GmHMGR4* 1-15 lines and *OE-GmHMGR6* 1-15 lines. M: DNA maker; B: blank control; N: negative control; P: positive control.

1.2 Supplementary Tables

Supplementary Table S1. Primers sequences of real-time fluorescent quantitative PCR

Gene	Forward primer (5′-3′)	Reverse primer (5′-3′)
Bar	GAAGGCACGCAACGCCTACGA	CCAGAAACCCACGTCATGCCA
GmACTIN	GGTGGTTCTATCTTGGCATC	CTTTCGCTTCAATAACCCTA
GmDXR1	TTAGTGTAGTCACAGGAATAGTAGGC	GCAAGAGGAAGAACAAAAGGAC
GmDXR2	CCCTGATGCCACCACTGTAG	ATGTTCCGAATCAGCGGGAA
GmHMGR1	GAAAAATCTTACTGGGTCTGCTATG	CTGATTGAGACGCCAGTTGTG
GmHMGR2	TGCTGGTTCTGCTGTCGC	CAAGTAAATTCAAGCAAGCAGATTA
GmHMGR3	TTTCTCCAGAGTGATTTTCC	CAGACCCAGTAAGGTTTTTC
GmHMGR4	TTCTTCAGAGTGACTTCC	GTCTTCAACACCTTCTTC
GmHMGR5	TTCTTCGGCATCCACTTCGTCCAG	GCTGTGCGTCTCCTTCTATGATGG
GmHMGR6	AAGCCGCAGCCGTGAATTGG	CCACCAAGAGCACCAGCCATG
GmHMGR7	TTCGGACAAGAAACCTGC	AAACCCACCAAGAGCACC
GmHMGR8	GCCGTTGTTGTTGGATGG	GCAATCCCTCAAAACCACA
GmFPPS	ATGGATAACTCTCACACACGC	TTCCCCTGAAGTGCTTTTTA
GmSQS	CCATAGCTGAACAGAAGAAGTCAG	CGCTGCGATCAAATTTCGATAACC
GmSQE	ATGTACGAATGGAGCAGGGC	AAACCCACGAAACAGGAGGG
GmCAS	AGGCAACAGGTCTCATGTGG	GGTATTCTCCCAACGCCCAA
Gmβ-AS	TGTATGCTTGCTTGTTGGGTT	GCAAAACCAGCATCCCATT
